# Safety and Efficacy of Radiofrequency Ablation in Management of Various Pancreatic Neoplasms

**DOI:** 10.3390/jcm14113958

**Published:** 2025-06-04

**Authors:** Varshita Goduguchinta, Mohamed Ebrahim, Raahi Patel, Navkiran Randhawa, Ahamed Khalyfa, Mahnoor Inamullah, Rahil Desai, Kamran Ayub

**Affiliations:** 1 Franciscan Health, Olympia Fields, IL 60461, USA; vgoduguchinta1@gmail.com (V.G.); raahip07@gmail.com (R.P.); rahildesai@gmail.com (R.D.); 2 Ascension Saint Joseph Hospital, Chicago, IL 60657, USA; mohdaymanebrahim@gmail.com; 3 Department of Gastroenterology, Medical College of Georgia, Augusta, GA 30912, USA; randhnk@gmail.com; 4 Department of Gastroenterology, The University of Iowa, Iowa City, IA 52242, USA; akhalyfa1@gmail.com; 5 Southwest Gastroenterology, Division of GI Partners of Illinois, Oak Lawn, IL 60453, USA; mahnoor.fm93@gmail.com; 6 Silver Cross Hospital, New Lenox, IL 60451, USA

**Keywords:** radiofrequency ablation, pancreatic neoplasms, pancreatic adenocarcinoma, pNET, intraductal papillary mucinous neoplasm

## Abstract

**Background/Objectives:** Pancreatic neoplasms, including adenocarcinoma, pancreatic neuroendocrine tumors (pNETs), intraductal papillary mucinous neoplasms (IPMNs), and high-grade cystic lesions, often require surgical resection as a form of curative treatment. However, comorbidities and high-risk features may preclude surgery. Endoscopic ultrasound-guided radiofrequency ablation (EUS-RFA) has emerged as a minimally invasive alternative with proven cytoreductive efficacy in solid tumors. This case series evaluates the safety and efficacy of EUS-RFA in patients with various unresectable, non-metastatic pancreatic neoplasms. **Methods:** A retrospective review was conducted on eight patients who underwent EUS-RFA at our institutions between July 2021 and February 2025. All patients were deemed unsuitable surgical candidates due to comorbidities such as advanced age, cardiovascular disease, renal insufficiency, and COPD or due to patient resistance to surgical intervention. EUS-RFA was performed using a 19-gauge RFA needle (Taewoong Corporation). Follow-up imaging was conducted 3 to 6 months after the completion of RFA treatment. **Results:** All eight patients demonstrated a good to excellent response in terms of tumor size reduction. The most notable response was observed in a patient with pNET, resulting in complete resolution from 15.6 × 12.0 mm to 0.0 × 0.0 mm after two RFA treatments. Other neoplasms, including pancreatic adenocarcinoma and intraductal papillary mucinous neoplasms (IPMNs), also demonstrated significant reductions. Mild post-procedure complications, including pancreatitis and abdominal pain, were noted in three cases. **Conclusions:** EUS-RFA is a promising alternative for managing unresectable pancreatic neoplasms in high-risk patients. Our findings support its use across various tumor types with favorable outcomes and minimal complications, reinforcing its role in expanding therapeutic options beyond surgery.

## 1. Introduction

Pancreatic cancer is the third leading cause of cancer-related deaths in the United States, and its mortality rates are continuing to rise [1]. The aggressive nature of this disease is due to rapid tumor growth, early local invasion, and a tendency for distant metastases. Additionally, pancreatic cancer often presents with vague and nonspecific symptoms, such as generalized abdominal pain in the right upper quadrant, unintentional weight loss, a loss of appetite, and jaundice. These symptoms are frequently overlooked or misattributed to benign conditions. As a result, many patients are diagnosed only after the cancer has progressed to advanced or metastatic stages, limiting their treatment options and worsening their prognosis.

Although significant advances have been made in systemic chemotherapy, targeted therapies, and surgical techniques, the overall survival outcomes for pancreatic cancer remain poor. The 5-year survival rate for pancreatic cancer remains the lowest of any cancer, at <5%, with a median survival of <6 months [2]. Standard treatment regimens, such as FOLFIRINOX and gemcitabine/nab-paclitaxel, have only modestly improved median survival rates. Surgical interventions, such as the Whipple procedure and pancreatic resection, have improved therapeutic options for pancreatic cancer. But only one-fifth of pancreatic cancer patients have resectable disease [3]. Additionally, these surgical options are limited to patients without significant comorbidities. Even with multimodal treatment approaches, disease recurrence is common, highlighting the need for novel therapeutic strategies applicable to a wide range of pancreatic cancer patients.

Given the limitations of traditional therapies for pancreatic cancer, novel approaches like radiofrequency ablation (RFA) have emerged as promising adjuncts to treatment. RFA is a minimally invasive technique that utilizes thermal energy to induce tumor necrosis and has demonstrated efficacy in reducing the number of tumor cells (cytoreduction) in various solid cancers, including liver, kidney, and lung cancer [4]. Previous studies, including those by Kim et al., have demonstrated the feasibility and safety of RFA in the porcine pancreas [5]. Its potential applications in pancreatic cancer offer a minimally invasive option for patients with unresectable disease. Furthermore, early evidence suggests that RFA may trigger systemic immunomodulatory effects, potentially transforming the tumor into an in situ vaccine and enhancing the effectiveness of concurrent therapies [6]. Integrating RFA into existing treatment algorithms for various pancreatic neoplasms is crucial for improving survival rates and broadening the therapeutic landscape for this aggressive cancer.

### Objective

With our case series, we wanted to evaluate the efficacy of EUS-RFA as an alternative to surgery in patients with various unresectable non-metastatic pancreatic neoplasms: pancreatic neuroendocrine tumors (pNETs), including insulinoma, adenocarcinoma, intraductal papillary mucinous neoplasm (IPMN) with a malignant nodule, and high-grade dysplastic pancreatic cysts and to report any adverse events experienced with this treatment modality.

## 2. Methods

We conducted a comprehensive retrospective analysis of all patients who underwent EUS-RFA for pancreatic neoplasms at our medical centers between July 2021 and February 2025, ensuring the inclusion of a sufficiently long follow-up period to reliably evaluate both short- and long-term treatment efficacy and safety outcomes. Patient selection was based on strict inclusion criteria, requiring histologically confirmed pancreatic neoplasms through EUS-FNA, with specimens reviewed by dedicated gastrointestinal pathologists, along with unresectable neoplasm due to either severe or multiple medical comorbidities contraindicating major surgery or the explicit refusal of surgical resection following thorough counseling by a multidisciplinary tumor board comprising experts in surgical oncology, gastroenterology, medical oncology, radiology, and pathology. Our final cohort comprised eight patients with diverse pancreatic neoplasms, including neuroendocrine tumors, adenocarcinomas, intraductal papillary mucinous neoplasms (IPMNs) with malignant features, and cystic lesions with high-grade dysplasia, all deemed poor surgical candidates due to factors such as advanced age (with a median of 80 years; range 41–91), cardiovascular disease, liver disease, chronic kidney disease, and severe pulmonary compromise. Notably, one patient with technically operable disease declined both surgery and systemic chemotherapy, electing instead for endoscopic treatment only, and was included in the cohort on the basis of informed patient preference aligned with study criteria.

All EUS-RFA procedures were performed in a dedicated endoscopy suite by an experienced therapeutic endoscopist with expertise in interventional EUS, utilizing monitored anesthesia care (MAC) with propofol for sedation in seven cases, while one patient with significant cardiopulmonary risk factors underwent general anesthesia with endotracheal intubation. We employed a linear-array therapeutic echoendoscope (Olympus Medical Systems, Tokyo, Japan), offering high-resolution imaging and Doppler capabilities. After the meticulous EUS characterization of each target lesion, including a Doppler assessment to map adjacent vasculature and minimize vascular injury risk, a 19-gauge RFA needle electrode (Taewoong Corporation, Busan, Republic of Korea) was used, which was specifically designed for the endoscopic ablation of pancreatic lesions. This needle electrode system features several key technological innovations that make it particularly suitable for the precise, controlled ablation of pancreatic neoplasms. The device consists of a sharp, insulated 19-gauge needle with an exposed active tip available in different lengths, including 5 mm, 7 mm, or 10 mm, allowing the endoscopist to select the appropriate ablation zone size based on lesion dimensions. The needle incorporates a dual-channel design that enables the simultaneous delivery of radiofrequency energy and continuous internal cooling with a chilled (0 °C) saline solution, which serves multiple critical functions. The cooling mechanism prevents charring and tissue adherence to the electrode tip during energy delivery, maintaining consistent impedance throughout the ablation while allowing for higher power settings without excessive carbonization that could limit energy penetration. The needle connects to a proprietary radiofrequency generator that provides real-time impedance monitoring and automated power adjustment, with the system capable of delivering up to 50 watts of ablation power as needed for different lesion types. The needle’s unique tapered design and sharp distal tip facilitated precise penetration into firm pancreatic tissue while minimizing trauma to the surrounding parenchyma. Its 19-gauge diameter provided an optimal balance between rigidity for accurate tumor targeting and flexibility for maneuvering through the echoendoscope’s working channel. The electrode’s bipolar design allowed controlled, localized energy delivery with minimal current dispersion, reducing the risk of collateral damage to adjacent structures.

Radiofrequency energy was delivered according to the protocol, with energy parameters, with adjustments made based on lesion size, type, and anatomical considerations, and tailored to the following lesion characteristics: smaller solid tumors (typically <15 mm) were treated with a 5 mm active tip electrode at 10 watts of power, while larger lesions (>15 mm) utilized a 7 mm tip at 20 watts, and cystic lesions with mural nodules received higher energy delivery at 50 watts. During ablation, the system’s real-time impedance monitoring capability provided objective feedback on treatment completeness, with characteristic impedance curves guiding the endpoint of each application (typically 400–500 ohms), indicating adequate thermocoagulation. Larger or irregularly shaped neoplasms required multiple overlapping applications (a median of 3 per session, in the range of 2–5) to ensure complete coverage, with careful repositioning under EUS guidance to avoid geographic misses. Immediate post-procedure monitoring in a recovery unit for 1–3 h included serial vital sign assessments and laboratory evaluation for pancreatitis. All patients underwent a CT of the abdomen/pelvis at 3–6 months intervals to assess treatment response, with repeat RFA sessions performed if a residual tumor was identified, employing identical technical parameters. This uniform procedural framework ensured technical consistency across the cohort while allowing case-by-case individualized adjustments based on tumor biology, anatomic challenges, and patient-specific risk factors.

## 3. Results

The clinical course and therapeutic outcomes of patients undergoing RFA for pancreatic lesions demonstrated both the efficacy and safety profile of this minimally invasive intervention. The aggregate outcomes across our entire case series of eight patients revealed several important trends regarding the application of RFA to pancreatic lesions. Our cohort demonstrated a median age of 80 years (a range of 41–91 years) with an equal gender distribution (four male and four female patients), reflecting the typical demographic profile of patients with pancreatic neoplasms. A quantitative analysis of tumor dimensions showed a median baseline measurement of 12.7 × 10.3 mm (range 11.0 × 8.5 mm to 25.0 × 20.0 mm) across all lesion types, excluding cystic lesions. Following RFA intervention, we observed a median post-procedural tumor size reduction to 5.5 × 4.0 mm (range 0.0 × 0.0 mm to 15.5 × 12.5 mm), representing a median volumetric reduction exceeding 50% from baseline measurements. This degree of tumor response was particularly notable given that all patients in our series were either poor surgical candidates or had declined operative management.

A case from our series involved a 75-year-old male patient with multiple comorbidities, including hypertension, hyperlipidemia, coronary artery disease, and iron deficiency anemia, who initially presented to our institution with persistent, dull abdominal pain localized to the epigastric region. Diagnostic evaluation commenced with contrast-enhanced CT imaging of the abdomen and pelvis, which revealed a subtle, hypodense mass lesion measuring approximately 15.6 × 12.0 mm in the distal pancreatic tail. This finding prompted further characterization via endoscopic ultrasound (EUS), which confirmed the presence of a well-circumscribed, hypoechoic nodule with homogenous internal echotexture and increased echotransmission, suggesting hypervascularity. EUS-guided fine needle aspiration (FNA) was subsequently performed, with cytopathological analysis confirming the diagnosis of a well-differentiated pancreatic neuroendocrine tumor (pNET). Given the patient’s advanced age, significant cardiopulmonary comorbidities, and high surgical risk as determined by our multidisciplinary tumor board, along with the patient’s strong preference against major pancreatic resection, RFA was selected as the optimal therapeutic approach. Using EUS guidance, a 19-gauge RFA needle with a 5 mm active tip was precisely positioned within the target lesion. Ablation was performed at 10 watts of power with a total of six overlapping applications to ensure complete coverage of the 15.6 × 12.0 mm tumor. The patient tolerated the procedure well and was monitored in the recovery unit for four hours post-procedure before being transferred to the medical floor. Follow-up imaging at three months post-ablation demonstrated complete radiologic resolution of the previously visible lesion, with only a small area of fibrotic scar tissue remaining at the treatment site, with the resolution of the tumor at a size of 0.0 × 0.0 mm after two RFA treatments (Table 1). However, this patient did experience a mild episode of post-procedural pancreatitis, as evidenced by the transient elevation of serum lipase to 850 U/L and findings on CT imaging, requiring three-day hospitalization for pain management and intravenous hydration. This complication was resolved completely with conservative measures, and the patient was discharged home in stable condition with instructions for close outpatient follow-up.

The neuroendocrine tumor subgroup (*n* = 2) demonstrated particularly robust responses to RFA therapy. Beyond the index case described above, our second pNET patient, a 76-year-old female with a 12.0 × 10.5 mm lesion in the pancreatic head, underwent two separate RFA sessions spaced 10 and 12 weeks apart, respectively. Each session employed a 19-gauge needle with a 5 mm active tip at 10 watts of power, with the first session consisting of three applications and the second session utilizing two applications. Follow-up imaging revealed a significant reduction in tumor size to 9.0 × 8.2 mm, representing an approximately 30% volumetric reduction, with no procedure-related complications.

Malignant pancreatic lesions, including adenocarcinoma and intraductal papillary mucinous neoplasms (IPMNs) with high-grade dysplasia or malignant transformation, also showed meaningful responses to RFA therapy. The adenocarcinoma case in our series involved a 91-year-old male with a 25.0 × 20.0 mm lesion in the pancreatic body. This patient underwent two RFA treatments spaced 12 weeks apart, with the first session utilizing a 19-gauge/7 mm needle at 20 watts of power for four applications and the second session employing a 19-gauge/5 mm needle at 10 watts for three applications. Follow-up imaging demonstrated a substantial reduction in tumor size to 15.5 × 12.5 mm, representing approximately 50% volumetric reduction. While this patient did experience moderate abdominal pain post-procedure, requiring opioid analgesia for 1 week, there were no other complications, and the patient reported significant improvement in his cancer-related pain symptoms at subsequent follow-up visits. The IPMN cases in our series (*n* = 2) showed particularly impressive responses to RFA therapy. The first session involved an 83-year-old female with a 19.0 × 10.0 mm malignant nodule within a pancreatic head cyst. She underwent a single RFA session using a 19-gauge/7 mm needle at 50 watts of power for three applications, resulting in near-complete resolution of the mural nodule (final size 9.0 × 6.0 mm) with no procedural complications. The second IPMN patient, an 80-year-old female with a 12.0 × 11.0 mm nodule within a larger 29.0 × 26.0 mm pancreatic cyst, was treated with a single RFA session using identical parameters (19-gauge/7 mm needle at 50 watts) but with only two applications. This resulted in a marked reduction in the nodule to 2.0 × 2.0 mm, though the patient did develop mild pancreatitis requiring two-day hospitalization. Both IPMN patients showed no evidence of nodule regrowth at six-month follow-up imaging. In an 87-year-old male diagnosed with pancreatic carcinoma, radiofrequency ablation (RFA) was performed using a 19-gauge, 5 mm needle at an energy setting of 10 watts. The procedure involved three targeted applications. Following treatment, the tumor size decreased from an initial measurement of 12.0 × 9.0 mm to 11.0 × 9.0 mm, and an approximately 8.3% reduction in the tumor area following treatment. The patient tolerated the procedure well, with no reported complications or adverse effects.

**Figure 1:**
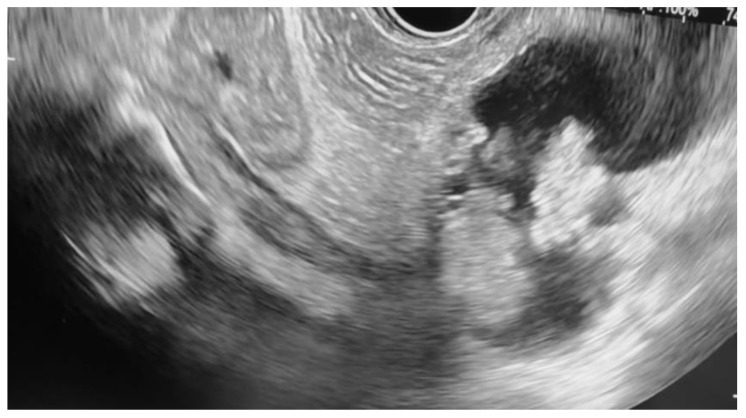
Pancreatic cyst with mural nodule and high-grade dysplasia on EUS.

**Figure 2:**
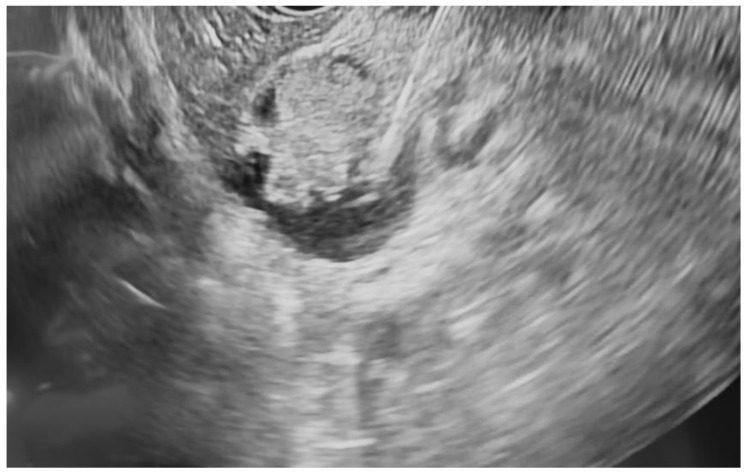
Pancreatic cyst fluid aspiration prior to RFA.

**Figure 3:**
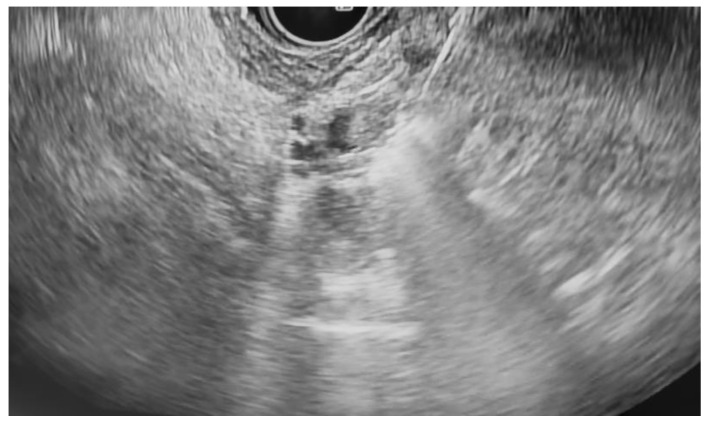
The RFA of the pancreatic mural nodule shown in Figure 1 and Figure 2.

**Figure 4:**
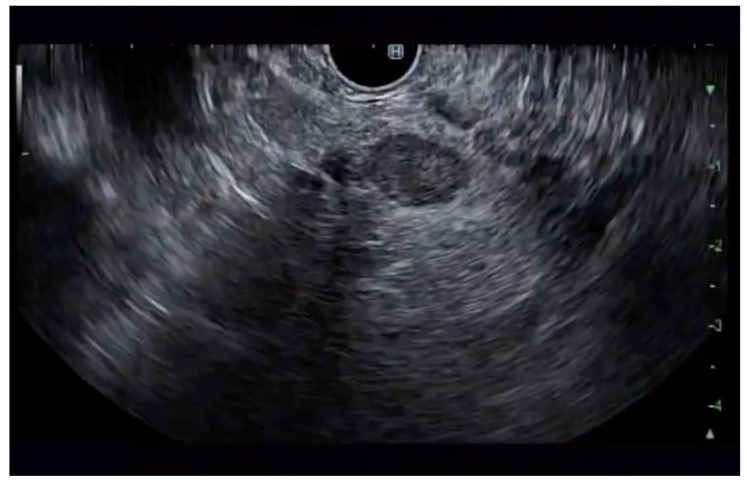
pNET at the neck of the pancreas (insulinoma) on EUS.

**Figure 5:**
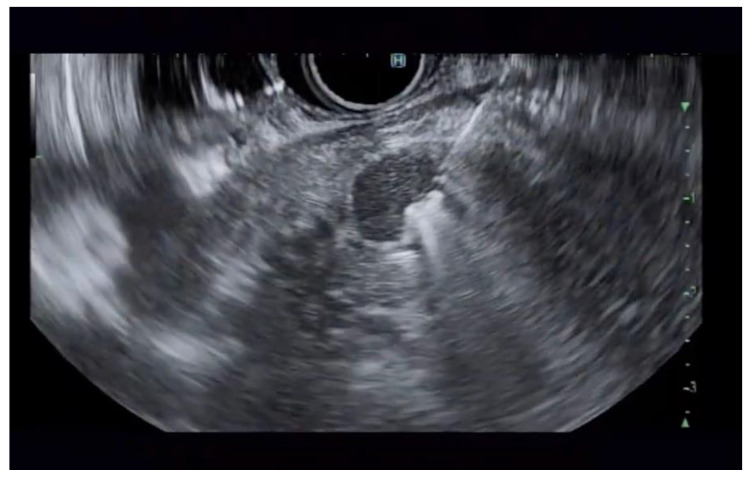
RFA application to the superior aspect of the insulinoma.

**Figure 6:**
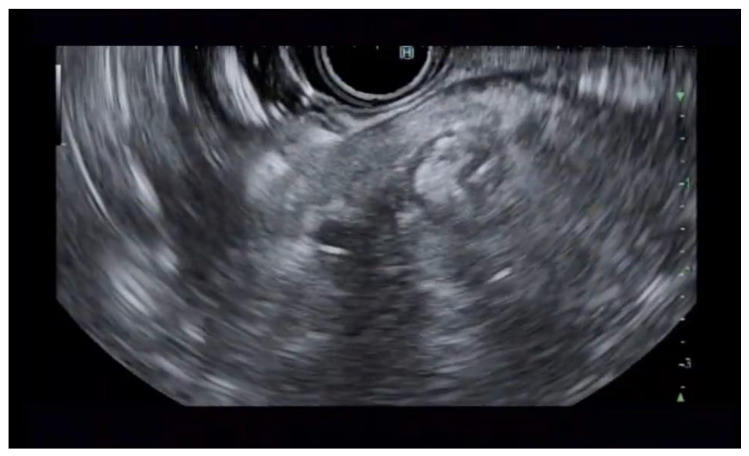
RFA application to the inferior aspect of the insulinoma.

Additional notable cases in our series included a pancreatic cyst with a high-grade dysplastic nodule in an 80-year-old female patient. The initial 11.0 × 10.0 mm nodule was completely ablated (0.0 × 0.0 mm residual) after a single RFA session using a 19-gauge/7 mm needle at 50 watts of power for three applications with no complications (Figure 1, Figure 2 and Figure 3). Similarly, a 41-year-old male with a 13.5 × 8.5 mm insulinoma in the pancreatic neck who presented with multiple syncopal episodes secondary to hypoglycemia achieved complete radiologic and biochemical resolution after a single RFA session using a 19-gauge/5 mm needle at 10 watts of power for two applications (Figure 4, Figure 5 and Figure 6), with no adverse events and normalization of his insulin and glucose levels post-procedure and the resolution of his syncope.

The safety profile of pancreatic RFA in our series was generally favorable, with no instances of infection, perforation, hemorrhage, or other major complications. The two cases of pancreatitis (25% incidence) were both mild and managed conservatively with brief hospitalizations. Procedural parameters were standardized using 19-gauge RFA needles throughout, with variations in active tip length (5 mm or 7 mm), power settings (10–50 watts), and the number of applications (2–5 per session) based on lesion characteristics and location. All procedures were performed by experienced interventional endoscopists with expertise in therapeutic EUS, contributing to the favorable outcomes observed in this challenging patient population. A detailed summary of the results is presented in Table 1.

## 4. Discussion

Pancreatic neoplasms are tumors that can develop within the pancreas and are classified based on two types: exocrine versus endocrine. While exocrine pancreatic neoplasms, such as adenocarcinoma, make up over 95% of pancreatic cancers, endocrine pancreatic neoplasms, such as neuroendocrine tumors, make up less than 5% [7]. While the primary treatment modality for pancreatic neoplasms remains surgical resection and is curative in early-stage tumors, it is associated with significant short and long-term adverse events. Some of these include the formation of pancreatic fistulas, delayed gastric emptying, postoperative hemorrhage, wound dehiscence, and more [8].

Examples of exocrine pancreatic neoplasms include pancreatic adenocarcinoma, acinar cell carcinoma, squamous cell carcinoma, intraductal papillary mucinous neoplasms, and mucinous cystic neoplasms, among others. Endocrine pancreatic neoplasms, commonly referred to as neuroendocrine tumors, encompass a range of conditions, including insulinomas, gastrinomas, glucagonomas, VIPomas, somatostatinomas, and others. They account for approximately 2% of all malignancies in the United States [9]. While exocrine neoplasms of the pancreas are more likely to become cancerous, endocrine neoplasms of the pancreas have the potential to become malignant, albeit at a much lower rate, and tend to have a better prognosis. Altogether, pancreatic cancer is the fourth leading cause of death in the United States of America [10]. Between 1990 and 2017, the number of pancreatic cancer cases doubled from 196,000 to 441,000 [11]. The key modifiable risk factors of pancreatic cancer include smoking cigarettes, alcohol use, type 2 diabetes mellitus, and obesity. With all the different types of neoplasms discussed above, the mainstay of treatment for all still remains surgical resection. Some of the various surgical options include pancreaticoduodenectomy (Whipple surgery), central pancreatectomy, distal pancreatectomy, and total or subtotal pancreatectomy. However, only 20% of pancreatic cancers are resectable at the time of diagnosis, with operative mortality rates of 1% to 16% [3]. Additionally, the majority of pancreatic cancers typically have a poor prognosis due to a high incidence of local and distant neoplasm recurrence. This brings forward the question about the role of radiofrequency ablation via endoscopic ultrasound for the management of unresectable pancreatic cancers.

A novel and emerging therapeutic approach for pancreatic neoplasms is EUS-RFA, as mentioned above [12]. Endoscopic ultrasound-guided radiofrequency ablation (EUS-RFA) is associated with significantly fewer adverse effects compared to surgical resection, as highlighted by Crinò et al. and our case series of eight patients, making it the preferable intervention for pancreatic neoplasms [13].

Interventional gastroenterologists perform this procedure using EUS to visualize and target the lesion. Treatment involves puncturing the tumor and delivering alternating currents via a specialized electrode at the needle’s tip. The resulting vibratory motion generates heat, causing localized disruption and coagulation necrosis of the tumor tissue, making it a viable alternative when surgery is not an option. Song et al. demonstrated the successful application of EUS-RFA in six patients with unresectable pancreatic cancer [14]. Pai et al., who conducted one of the first pilot studies on the use of EUS-RFA for pancreatic cystic lesions, demonstrated complete resolution or a 50% reduction in lesion size without any adverse effects in six patients [15]. In addition, Rossi et al. showed that 10 patients diagnosed with neuroendocrine tumors achieved successful ablation, with no recurrence of symptoms, at a median follow-up of 34 months [16]. Multiple studies have demonstrated that EUS-RFA is not limited to a single type of pancreatic lesion but rather has the ability to successfully treat various forms of pancreatic lesions similar to those seen in our case.

Moving forward, several areas warrant further investigation to optimize the role of EUS-guided RFA in pancreatic neoplasms. Prospective, multicenter studies with larger patient cohorts are needed to validate our findings and to better characterize predictors of treatment success and risk factors for complications. Stratifying outcomes by tumor type, size, location, and proximity to critical vascular structures would provide critical insights into which patients derive the greatest benefit from RFA and how procedural parameters can be tailored accordingly [17]. Additionally, standardized RFA protocols, including optimal energy settings, needle selection, and ablation duration, must be established to reduce variability in outcomes across different centers.

Another important development involves exploring the integration of EUS RFA with other therapeutic modalities. Preclinical and early clinical data suggest that tumor ablation may expose tumor antigens and prime an anti-tumor immune response [18]. RFA has demonstrated synergistic potential when combined with immunotherapy in hepatocellular carcinoma (HCC), where it enhances anti-tumor immunity and counters immunosuppression [19]. However, translating this strategy to pancreatic cancer faces unique challenges due to the inherently immunosuppressive tumor microenvironment characterized by dense stroma, poor immune cell infiltration, and low immunogenicity. While incomplete RFA (iRFA) in HCC risks triggering aggressive recurrence through proliferative and angiogenic pathways (often mitigated by adjuncts such as metformin, hydroxychloroquine, sorafenib, bevacizumab, CTLA-4 inhibitors, or interferon-α that may help mitigate these effects [19]), pancreatic tumors demand more nuanced approaches. Early evidence suggests that RFA may still inform immune responsiveness in pancreatic lesions when combined with stromal-modulating agents or checkpoint inhibitors [20]. These strategies remain in the experimental phase and require more evidence before becoming standard practice. Thus, combining RFA with systemic therapies such as immune checkpoint inhibitors, chemotherapy, or novel targeted agents holds considerable promise in enhancing treatment efficacy, particularly for aggressive subtypes like pancreatic adenocarcinoma [21]. Yet, key aspects like the optimal timing and sequence of treatments, drug selection, and the role of patient-specific factors such as tumor size and location require further research. The complexity of these variables underlines the importance of personalized treatment plans. Large-scale clinical trials are needed to clarify best practices, including patient selection, treatment cycles, dosage standards, and predictive biomarkers for response. Furthermore, longitudinal studies evaluating long-term oncologic outcomes, including disease-free survival, overall survival, and the impact on quality of life, will be critical to firmly establishing the role of EUS RFA within the broader treatment algorithm for pancreatic cancer.

## 5. Conclusions

In this case series, we demonstrate that the EUS-RFA of pancreatic neoplasia is a viable option for selected patients who are not candidates for pancreatic surgery. Specifically, this case series demonstrates that it is an excellent choice for a diverse range of pancreatic lesions, with a favorable safety profile and an efficacious response in patients with lesions, including pancreatic adenocarcinoma, neuroendocrine tumors, and pancreatic cysts.

## Figures and Tables

**Table 1:** Clinical Characteristics and Treatment Outcomes of Patients Undergoing EUS-Guided Radiofrequency Ablation for Pancreatic Lesions. The table details patient profiles, initial lesion dimensions, number of RFA sessions, technical parameters used during ablation, and post-treatment lesion size. Complications following RFA are also reported to assess the safety and effectiveness of the procedure across various pancreatic tumor types.

Patient Diagnosis	Patient Demographics	Initial Neoplasm Size	# of RFA Treatments	Application of RFA		Final Neoplasm Size	Complications
Neuroendocrine Tumor	75 yo M	15.6 × 12.0 mm	2	19-gauge 5 mm needle 10 watts 5 applications	19-gauge 5 mm needle 10 watts 4 applications	0.0 × 0.0 mm	Pancreatitis
Neuroendocrine Tumor	76 yo F	12.0 × 10.5 mm	2	19-gauge 5 mm needle 10 watts 3 applications	19-gauge 5 mm needle 10 watts 2 applications	9.0 × 8.2 mm	None
Adenocarcinoma	91 yo M	25.0 × 20.0 mm	2	19-gauge 7 mm needle 20 watts 4 applications	19-gauge 5 mm needle 10 watts 3 applications	15.5 × 12.5 mm	Abdominal Pain
Intraductal Papillary Mucinous Neoplasm with Malignant Nodule	83 yo F	19.0 × 10.0 mm	1	19-gauge 7 mm needle 50 watts 3 applications	N/A	9.0 × 6.0 mm	None
Intraductal Papillary Mucinous Neoplasm with Malignant Nodule	80 yo F	12.0 × 11.0 mm; Cyst: 29.0 × 26.0 mm	1	19-gauge 7 mm needle 50 watts 2 applications	N/A	2.0 × 2.0 mm	Pancreatitis
Pancreatic Carcinoma	87 yo M	12.0 × 9.0 mm	1	19-gauge 5 mm needle 10 watts 3 applications	N/A	11.0 × 9.0 mm	None
Pancreatic Cyst with Nodule High-Grade Dysplasia	80 yo F	11.0 × 10.0 mm	1	19-gauge 7 mm needle 50 watts 3 applications	N/A	0.0 × 0.0 mm	None
Insulinoma	41 yo M	13.5 × 8.5 mm	1	19-gauge 5 mm needle 10 watts 2 applications	N/A	0.0 × 0.0 mm	None

## Data Availability

The original contributions presented in this study are included in the article. Further inquiries can be directed to the corresponding author (Kamran Ayub).

## Abbreviations

The following abbreviations are used in this manuscript:

IPMN	Intraductal papillary mucinous neoplasm
pNET	Pancreratic neuroendocrine tumors
RFA	Radiofrequency ablation
EUS	Endoscopic ultrasonography
EUS-FNA	Endoscopic ultrasonography fine-needle aspiration biopsy
